# Personality-Based Affective Adaptation Methods for Intelligent Systems

**DOI:** 10.3390/s21010163

**Published:** 2020-12-29

**Authors:** Krzysztof Kutt, Dominika Drążyk, Szymon Bobek, Grzegorz J. Nalepa

**Affiliations:** Jagiellonian Human-Centered Artificial Intelligence Laboratory (JAHCAI) and Institute of Applied Computer Science, Jagiellonian University, 31-007 Krakow, Poland; dominika.a.drazyk@gmail.com (D.D.); szymon.bobek@uj.edu.pl (S.B.); gjn@gjn.re (G.J.N.)

**Keywords:** affective computing, adaptation, emotion detection, personality assessment, wearable sensors

## Abstract

In this article, we propose using personality assessment as a way to adapt affective intelligent systems. This psychologically-grounded mechanism will divide users into groups that differ in their reactions to affective stimuli for which the behaviour of the system can be adjusted. In order to verify the hypotheses, we conducted an experiment on 206 people, which consisted of two proof-of-concept demonstrations: a “classical” stimuli presentation part, and affective games that provide a rich and controllable environment for complex emotional stimuli. Several significant links between personality traits and the psychophysiological signals (electrocardiogram (ECG), galvanic skin response (GSR)), which were gathered while using the BITalino (r)evolution kit platform, as well as between personality traits and reactions to complex stimulus environment, are promising results that indicate the potential of the proposed adaptation mechanism.

## 1. Introduction

Because technology is becoming more ubiquitous and pervasive, people interact with an increasing number of devices, like a range of intelligent appliances integrated with their home or office space. The widespread development of such Intelligent Systems (IS) used by individuals requires not only intelligent methods for problem solving, but, more importantly, intelligent methods of the adaptation of these systems. Furthermore, there is a persistent need to design user interfaces that are not only functional, but also accessible and user-friendly. The new concept of natural user interfaces was proposed over two decades ago, in order to consider ones that would learn how the user engages with them and how they adapt to the user’s needs. Because people are anthropomorphic towards everything that they may interact with, e.g., “we verbally praise them when they do a good job for us or blame them when they refuse to perform as we had wished” [1], there is a need to incorporate information regarding user emotions into the adaptation, including the interface. This is particularly important in mobile and ambient systems, because they assist us during many activities. In general, the incorporation of emotion processing into intelligent systems can make them more natural and humanized in operation and interaction [2]. The development of such systems lies in the area of affective computing (AfC). It is an interdisciplinary field of study that provides a general framework for the development of methods, models, and tools that are widely related to the processing and use of data on human emotions in computer systems [3,4].

In our work, we are aiming at the development of practical technology for everyday use based on mobile and wearable devices. This assumption lead to the choice of context-aware systems (CAS) as the basis. Context is understood here as “any information that can be used to characterize the situation of a subject” [5], e.g., current Facebook posts stream, geospatial localization, movement speed, or calendar events. Most of the modern CAS solutions are developed for the use of mobile and wearable devices (e.g., smartphones or Internet of Things devices), where the use of many independent sensors is facilitated. They include various types of biomedical devices that can collect emotion-related physiological signals, like wristbands, and external DIY hardware platforms, such as Arduino, Raspberry Pi, or BITalino (for comparison of mobile electrocardiogram (ECG) and galvanic skin response (GSR) sensors see [6]). The contextual data in mobile CAS are usually fused from multiple sources, like mobile device sensors (such as GPS), third-party services (e.g., weather forecasts), and the users themselves (via the form of questionnaires or a feedback loop). In such a setting, the system needs to be able to cope with heterogeneous and vague or missing data. We previously defined four requirements for building such a system [7].

We have developed a framework that allows for us to meet the described requirements. Furthermore, we extended this framework to support affect-aware systems, leading to the creation of a general architecture of mobile platform for emotion recognition and processing that we call the “Affective Computing with Context Awareness for Ambient Intelligence (AfCAI)” framework (for a summary of the history of our approach, see [8,9]). In our framework, we follow James–Lange non-cognitive view on emotions, where they are understood as perceptions of bodily changes [10]. This theory was extended by Prinz [11] with a second element, specifically a relationship between the subject and environment. From the CAS systems’ point of view, we can treat this information as a context. For example, in a specific situation, a faster heart rate (a bodily change) and perception of a danger, e.g., a predator (a context), build up fear. The importance of the context in predicting emotions must be emphasized, e.g., the same smile may appear in many different situations and it may not necessarily always mean joy. We restrict ourselves to analysis of a limited set of bodily signals regarding heart activity and skin conductance, as we are aiming at the usage of cheap and affordable technology. In the general framework, these bodily changes can be complemented by any context. In addition, current affective state can be considered to be a part of context for the whole system.

The incorporation of affective information into IS can take various forms. On the one hand, there are studies in which models are trained on the whole data set and general associations are examined, e.g., “what are the differences in the ECG between low and high emotion intensity in the whole collected data sample?”. If one attempts to infer about the entire population in the study, it will take a form of a “classical” psychological methodology used, e.g., in personality science. However, the direction of development of modern affective technology is quite opposite. Researching how people, in general, react to a given stimulus is not the main focus of this paper. Instead, in our approach, we emphasize the need for the personalization of affective systems in order to meet the expectations and needs of individual users (for a current trends review at the intersection of personality science and personalized systems, see [2]). Therefore, the computational models of emotions need to be adaptable in order to reflect the individual differences.

Deciding on an affective adaptation mechanism requires a choice between a generic solution, which is easier to prepare, but less accurate from the user’s point of view, and a fully personalized model that is difficult to implement due to many issues, such as the need to collect large amounts of data and develop appropriate individual theories of mind. As a compromise between these two extremes, we propose using personality assessment as an aggregation mechanism, which allows for differentiating the behavior of the system, depending on different types of personality. Our hypothesis is that this psychologically-grounded mechanism will divide users into groups that differ in their reactions to complex emotional stimuli, which will allow for personality-based affective adaptation.

We use two kinds of proof-of-concept demonstrations of our approach in order to verify our assumptions. Firstly, we conducted “classical” experiments, in which we presented stimuli to the subjects and collected their answers: both with questionnaires and physiological signals measurement. Secondly, as a testbed, we developed simple computer games as a specific context, since they provide a controllable environment in which experiments can be easily carried out. Within this context, it is easy to manipulate incoming stimuli and collect information about the subject’s behaviors.

The rest of the paper is organized, as follows: we begin with short methodological introduction on selected methods for measuring emotions and their links with personality in Section 2. Subsequently, in Section 3, we discuss our experimental setup. The analysis of results starts with the validation of our own widgets for subjective assessment of emotions in Section 4. We then move on to the analysis of relationships between personality and affective content: simple audio-visual stimuli are discussed in Section 5, while complex environments (games) are addressed in Section 6. Section 7 concludes the paper.

## 2. Emotions and Personality in Intelligent Systems

### 2.1. Emotion-Related Data Collection

In our work, we will assume that the operation of an intelligent system changes depending on the user’s behavior. Our objective is to adjust the system’s actions to best suit users’ personality and mood. In order to do so, we assume that information regarding the emotional condition of the person can be measured, i.e., expressed both in quantitative and qualitative ways. Our perspective is an engineering one, as we evaluate and select methods from psychology and affective computing in order to deliver information that is needed for adaptation and, eventually, personalization.

Firstly, in order to measure emotions, an appropriate conceptualization is needed—it will indicate what specific measurements and under which conditions should be carried out. In our work, we follow the common approach of considering the two dimensional model of valence and arousal (an overview of emotional models that are used in human–computer interaction can be found in [12]). Valence differentiates states of pleasure and displeasure, while arousal contrasts states of low activation/relaxation and excitation [13,14]. These dimensions are revealed in the activity of the Autonomic Nervous System [15].

Emotions can be expressed in various ways. Therefore, there are many means of collecting data regarding affective states. It is crucial to note that physiological signals are one of the most important data to reflect emotions. These include information regarding heart, muscle, and brain, as well as respiration or skin sweating (for meta-analysis, see [16,17]). Several observable human behaviors should also be noted. Following Ekman’s paradigm [18], people express their emotions while using their faces. What is more, individuals do it unintentionally, and one can observe so-called micro-expressions, even if the person is determined to hide real emotions [19]. Furthermore, emotions are correlated with changes in postures, gestures, and prosody [17].

Another aspect of emotions is connected with cognition. People are able to observe changes that happen to them and interpret them in a certain way. Therefore, another method of gathering emotion-related data requires asking subjects about their emotional state. It can be done, i.e., after presentation of emotion-inducing stimulus [20] or after the set of such stimuli during an experiment [21]. What is more, people can be asked about specific discrete emotions [21] or about the intensification of the characteristic on a given dimension [22].

Finally, as the research results show, collecting data from various modalities does not always help to create better models, as correlations are mediocre at best. For example, the analysis of data that were collected in the DREAMER database [23] showed that the use of simultaneous EEG and ECG signals is just as effective as using either EEG or ECG alone. Subsequently, when creating emotional models while using different modalities, one should take proper fusion of signals [24] into account, as it can be carried out on different possible levels with different accuracy [25]. One of high-level artificial intelligence (AI) approaches to fuse information from different sources in order to infer about condition of an entity is the context-aware systems paradigm that we selected as a base (as introduced in Section 1).

It is not necessary to conduct one’s own investigations to get data related to emotions, as several research teams share data from their studies. One can choose a dataset that is appropriate to the current needs: carried out on professional equipment or wearables, in the form of a classic stimulus-reaction experiment, or a form of social interaction. In the case of an experiment, stimuli can be sounds, images, or videos. Social interactions can be in pairs or in larger groups. It is also possible to choose a dataset that has specific signals (e.g., EEG or ECG). The K-EmoCon dataset authors provide the up-to-date overview [26]. Unfortunately, these datasets suffer from a small research sample [26,27]. In review [26], only the SEMAINE study [28] has more than 100 participants, while the others have an average of 30 (minimum seven, maximum 64).

### 2.2. Personality Traits in Affect Recognition and Games

When considering the methods of assessing personality, the most relevant method is the five-factor model that was developed by Costa and McCrae [29]. In the so-called “Big Five” model, personality consists of five traits: Neuroticism, Extroversion, Openness to experience, Agreeableness, and Conscientiousness. Although there are several doubts, many studies indicate the universality of such a construct and its independence from culture, social status, and economic capabilities [30].

The interest in integrating personality assessment into affective computing is reflected in the creation of two publicly available data sets: ASCERTAIN [31] and AMIGOS [32]. They are the results of experiments in which simple emotional stimuli (pictures, sounds, movie clips) were presented to the subjects. Their physiological reactions (e.g., ECG and GSR signals) were collected and then combined with the “Big Five” assessment. The authors also presented the preliminary results on their data sets indicating the existence of significant relationships between personality factors and the characteristics of physiological signals. Further analysis of the ASCERTAIN collection led to the creation of a model that was based on hypergraph learning demonstrating the usefulness of personality assessment in the prediction of emotions [33].

The relationship between personality traits and games was also explored. Several findings indicate that changes in the personality profile are linked to preferences for different games’ genres [34,35,36]. For example, the preference for adventure games is correlated with agreeableness (ease of identification with game characters) and openness (preference for big complex game world). However, it should be noted here that these are not strong associations. The authors point to the complexity of the player–game interactions, which, apart from personality, is also influenced by friends and advertisements, current mood, motivation, and more [34,37]. The new studies use more advanced methods than just simple correlation, such as hierarchical clustering [38], indicating more complex associations.

The personality assessment does not have to involve a personality test. One of the possibilities is to use personality stories [39]—a robust and lightweight methodology that allows for a more ecological evaluation and one that could also be used in order to estimate other psychological characteristics. Another way is to use Automatic Personality Recognition (APR) methods considered in personality computing. It is the “task of inferring self-assessed personalities from machine detectable distal cues” [40]. The most common objective is to determine the “Big Five” personality traits on the basis of various types of behavioral clues, such as texts (essays, social media messages), non-verbal communication, cell phone usage logs, game activity, or wearables’ signals [40]. However, to the best of our knowledge, there are no public APR-related datasets available. These studies often have much larger numbers of subjects (even over 1000 people), although this is related to a more simplified methodology when compared to AfC. The APR studies do not collect such a large number of signals and, instead, focus on a very specific type of behavioural clues (e.g., only blog posts) [40].

## 3. Materials and Methods

We conducted an experiment consisting of two main parts in order to verify our hypothesis about the usefulness of personality profiles as a grouping mechanism for the effective adaptation of intelligent systems (see Section 3.1). In order to address the shortcomings of existing datasets (see Section 2.1), the presented study was carried out on more than 200 subjects to provide a bigger dataset to AfC community. Equally importantly, our dataset combines emotion-related data with contextual information. Finally, in our opinion, the presentation of longer stimuli, such as the movie clips that were used in two personality-related datasets (51–150 s in ASCERTAIN, 51–128 s in AMIGOS; see Section 2.2), makes later analysis difficult, as emotions may have changed many times during this interval. Therefore, in our study, we do not only compile psychophysiological signals with information of complex stimuli presented to the subjects. Our final dataset contains the results of the experimental phase with short stimuli, which can be used, e.g., for system calibration. Logs from games (a complex stimuli) complement this, which can be divided into detailed series of events (recorded in logs), making their analysis simpler than movie clips.

The data that were collected in the experiment have been processed, i.e., the physiological signals have been filtered, the images have been analyzed using MS API to recognize facial emotions, and the “Big Five” factors were calculated. The final version of the collected dataset, called BIRAFFE: Bio-Reactions and Faces for Emotion-based Personalization is publicly available at Zenodo under CC BY-NC-ND 4.0 license (http://doi.org/10.5281/zenodo.3442143) [41]. In the remainder of this section, key elements of the study design will be outlined. For detailed technical description of the dataset itself, see [42].

### 3.1. Study Design

The study was carried out on 206 participants (31% female) between 19 and 33 (*M* = 22.02, SD = 1.96; the statistics were calculated for 183 subjects for whom information about age and sex is included in the final dataset). Information regarding recruitment was made available to students of the Artificial Intelligence Basics course at AGH University of Science and Technology, Kraków, Poland. Participation was not an obligatory part of the course, although one could get bonus points for a personal participation or the invitation of friends.

In one part of the study, simple sound and visual stimuli from standardized affective stimulus databases were presented (see Section 3.3). Subjects evaluated emotions that were evoked by them using our two proof-of-concept widgets (see Section 3.4). In the second phase of the experiment, the subjects played two affective games that exposed them to complex emotional stimuli (see Section 3.5). During the whole experiment, the ECG and GSR signals were collected with the BITalino (r)evolution kit platform (https://bitalino.com) and the photos were taken with the Creative Live! Cam Sync HD 720p camera. The whole experiment was controlled by the Sony PlayStation DualShock 4 gamepad. In addition to the computer-based part, to measure the “Big Five” personality traits, the subjects filled in the paper-and-pen Polish adaptation [43] of the NEO Five Factor Inventory (NEO-FFI) [29].

The study was carried out in a designated room at the university. During the experiment, there were three people present in the room: the researcher and two participants. The subjects were sitting in front of the computer stands that were arranged at the opposite walls, i.e., they were sitting with their backs turned to each other. The instructions and explanations were presented to both subjects at the same time. During the procedure, the researcher was sitting at a separate desk with his or her back to the subjects in order to overcome Hawthorne effect.

### 3.2. Ethics Statement

The Research Ethics Committee of the Faculty of Philosophy of the Jagiellonian University reviewed the described study and it received a favourable opinion. Informed written consent was obtained from all of the participants.

### 3.3. Stimuli Selection

Standardized emotionally-evocative images and sounds from IAPS [44] and IADS [45] sets were used as stimuli. Both of the data sets are provided together with information on the emotional characteristics of each stimulus, written in the form of coordinates in the Valence–Arousal space.

**Figure 1:**
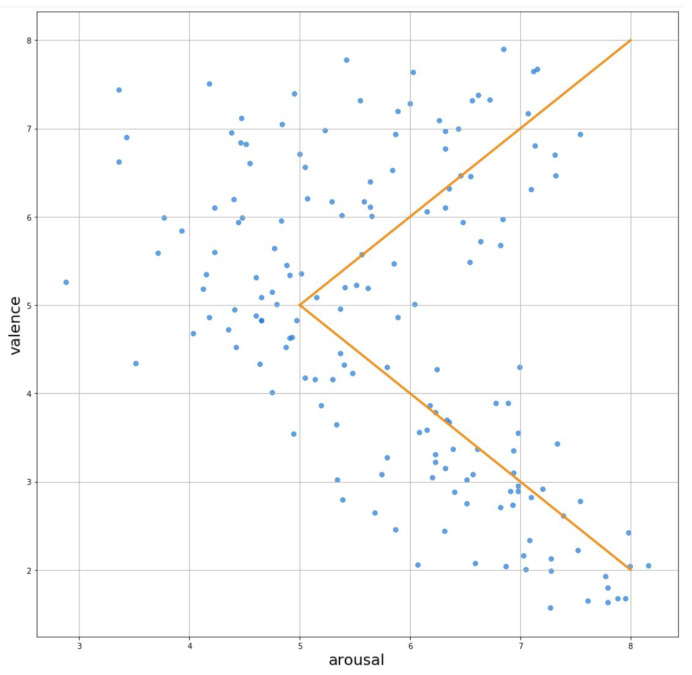
Trends in the IADS set stimuli ratings.

The analysis of IADS set sounds’ valence and arousal scores led us to the observation that there is a clear trend in this set: the arousal of emotions increases when the valence of sound is more extreme (positive or negative). Figure 1 depicts this observation. In the IAPS set, the Valence–Arousal space is better covered and we do not observe any trends. This is probably due to the fact that IAPS collection is much more numerous (167 sounds in IADS, 1194 pictures in IAPS).

For the purpose of the experiment, we divided the stimuli into three groups according to their arousal and valence index: + (positive valence and high arousal), 0 (neutral valence and medium arousal), and – (negative valence and high arousal). Afterwards, sounds and pictures were paired in two ways. First condition involved consistent types of pairs: + picture was paired with + sound (*p+s+*), 0 picture was paired with 0 sound (p0s0), and – picture was paired with – sound (*p–s–*). Second condition was inconsistent, composed of types: + picture matched with – sound (*p+s–*), – picture matched with + sound (*p–s+*).

Because we aimed to preserve equal proportion of pairs of each condition, and due to the different cardinality pair types inside each condition (three in consistent, two in inconsistent), the overall proportion of types of pairs presented itself, as follows: 20 for each type in consistent group, 30 for each in inconsistent; resulting in 120 stimuli for the whole experiment.

### 3.4. Emotion Evaluation Widgets

When considering the emotional assessment, the most widespread method is Self-Assessment Mankin (SAM) [22] evaluating the human emotional response to stimuli on three separate dimensions: arousal, valence, and dominance. Yet, it was reported [46,47] that, nowadays, SAM pictures lack clarity, especially due to the technology development. More fluent emotional assessment tools are better understood by users and they can more efficiently capture nuances of rating [47]. At the same time, modern affective rating tools that were developed for the purpose of continuous reaction assessment [48,49,50] did not meet the needs of our paradigm.

In order to maximize the amount of information, we could obtain and fit to the time constraints assumed in the experiment (nine second rating window); we decided to develop our own assessment tools. We also decided to exclude dominance dimension present in SAM, as this one was said to be the hardest to comprehend [51]. Finally, we proposed two widgets: “Valence-arousal faces” and “5-faces”. Both were controlled by a left joystick on a gamepad that was used by the participants in the experiment.

#### 3.4.1. Valence-Arousal Faces Widget

This widget is a composition of two state-of-the-art methods for emotion rating: Valence–Arousal space [52] and AffectButton [53]. The former gives a possibility to select a point on a two dimensional space, where one dimension ranges from negative to positive (valence), while second dimension—from low to high arousal. These dimensions are abstractive and difficult to use, as participants in our previous experiments indicated (see, e.g., [54]). In the latter case, AffectButton makes it easier to understand and reason about the options provided, since it uses emoticons as points of reference. That being said, its manipulation is not intuitive, and long training is required in order to know how to navigate towards the selected emotion.

**Figure 2:**
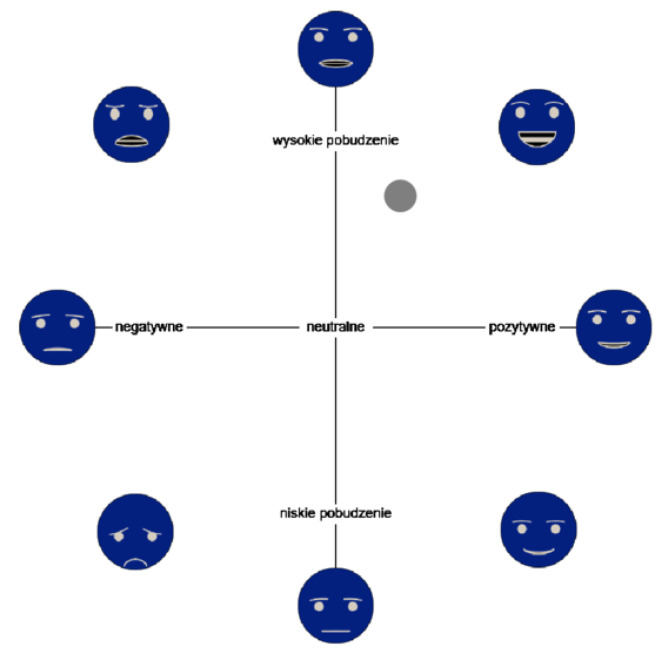
“Valence-arousal faces” widget as in the study (in Polish). *X* axis has labels “negative”, “neutral”, and “positive”, while the *Y* axis has labels: “high arousal” and “low arousal”. The picture is presented with a negative filter.

In our valence-arousal faces widget, there is a simple two dimensional space with ratings translated to [−1, 1] range, but, as a hint, we also placed eight emoticons from the AffectButton. These heads are placed on the most characteristic points of the space, i.e., −1 (the lowest score), 0 (neutral score), and 1 (the highest score). What is more, we used a simplified EmojiButton in response to the overly complex emoticons in the original AffectButton [55]. Figure 2 presents the final widget. It should be noted that our widget has both axes’ names and emoticons, which makes it different from EmojiGrid, where the authors replaced text with emoticons [47].

#### 3.4.2. 5-Faces Widget

**Figure 3:**
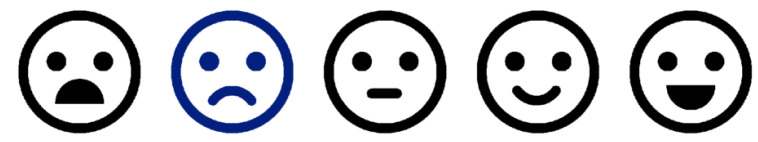
“5-faces” widget. The picture is presented with a negative filter.

This widget was introduced in order to provide a simple emotion evaluation tool. It consists of five emoticons (see Figure 3) that reflect the trend that was observed in IADS, i.e., the arousal is higher when the valence is more extreme (as depicted in Figure 1). Our intuition is that such a simple widget can give us enough information to address the hypotheses, and it is easier to use by the participants.

### 3.5. Games

Two affective games were used in the study. They were both designed and developed by our team. Both are fully controllable by a gamepad.

#### 3.5.1. Affective SpaceShooter 2

**Figure 4:**
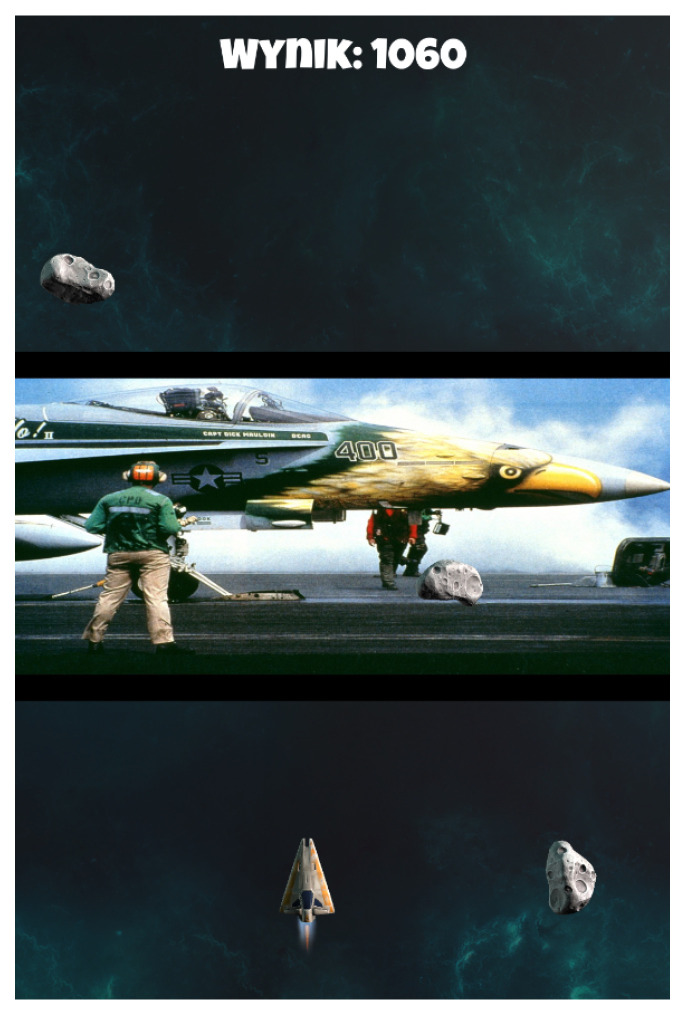
An example of the “Affective SpaceShooter 2” gameplay, asteroids falling, and an affective picture in the background [56].

The game is a variation of the classic Asteroids game in which the player controls a spaceship and its task is to shoot down or avoid floating obstacles. In the version used in the experiment, as in our previous prototype [9,56], the player’s ship is always at the bottom of the screen, and asteroids are coming from the top. The game uses two types of asteroids: grey (neutral) and colored (affective). Shooting down the latter causes the presentation of sound and visual stimuli (see Figure 4), according to the random assignment of color to one of the conditions: *p+s+, p–s+, p+s–, p–s–* (as described in Section 3.3). In the second half of the game, the stimuli are presented randomly (regardless of the color of the asteroid) in order to check the player’s reaction to the Inconsistent Reality Logic design pattern.

#### 3.5.2. Freud Me Out 2

**Figure 5:**
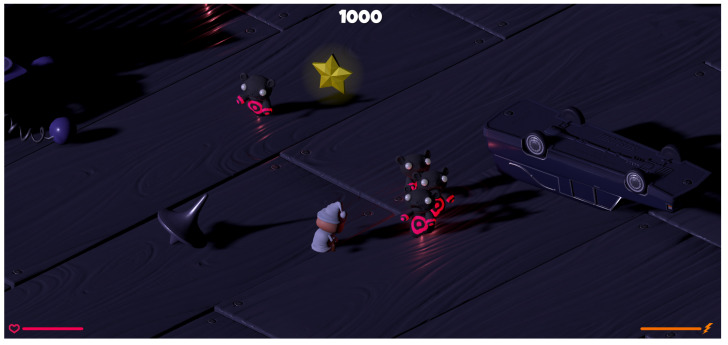
An example of the “Freud me out 2” gameplay [56].

In this isometric view game, which is the modification of our previous prototype [9,56], the player’s task is to defeat enemies—nightmares—and/or collect stars (see Figure 5). Players can freely combine these two types of activities, so long as they obtain the score that is required to complete each level. One can use both a regular handgun and a “SuperPower” to fight. The latter allows for attacking several creatures in a certain area around the protagonist at once. During the experiment, after the second of the five weeks of study, the maximum number of opponents was reduced from 30 to 12. As it turned out, the initial amount of creatures prevented the users from being able to choose a strategy of only collecting stars.

### 3.6. Analyses Overview

Over 100 variables that were related to all of the aforementioned elements of the procedure were collected in the study (stimuli presented, widgets’ responses, games’ logs, psychophysiological signals, and face emotions). For their detailed list and operationalization, see [42].

The remaining part of the paper provides the results of three investigations. Firstly, Section 4 provides the validation of two proposed widgets for emotion assessment. The next two sections refer directly to the main concern of this paper, i.e., verification of the usefulness of personality as a base for IS adaptation mechanism. In order to assess this, the relationships between emotions and personality were checked, revealing the potential for grouping individuals with similar characteristics of emotional responses (both self-assessment and physiological) using personality (see Section 5). Next, the relationship between the personality and actions taken in a rich (game) environment was investigated. The obtained relationships indicate the possibility of adapting this environment (and, ultimately, the IS) to different personality-based groups (see Section 6).

Statistical analysis was performed while using the R environment (version 3.5.3) [57] with lme4 library (version 1.1-23) [58] and MASS library (version 7.3-51.1) [59]. Models with the outcomes being numbers of occurrences of emoscale ratings clusters (A–P; defined in Section 4.1), as well as models with emospace ratings, were fitted while using Generalised Linear Mixed Models [60] with the Poisson distribution. Models with emospace responses (valence and arousal axes) as outcomes, as well as all of the models with biosignal responses as outcomes, were fitted using the Linear Mixed Models [60] with normal distribution. For all mixed models that are presented in the manuscript (in this and following sections), the visual inspection of residual plots, as well as other assumptions of fit, were checked and revealed no manifest deviations. The models were fitted by maximum likelihood Laplace Approximation [58] method. Deviance was obtained and compared for all models, each time by juxtaposing the full model, one including the fixed effect in question, with the model without the fixed effect in question, in order to establish the final structure of the random effects [61,62]:

(1) $$null\;model:y∼1+B_{0}+\left(B_{0}|random\;effects\right)+\epsilon_{i}$$

For the emoscale ratings that were clustered (A–P) as outcomes, multinomial logistic regression models were fitted by multinom function from nnet library (version 7.3-14) [59]. Deviance was obtained and compared for all models, each time by juxtaposing the full model, one including the fixed effect in question, with the model without the fixed effect in question.

## 4. Widgets Validation

In this study, two new widgets were proposed to assess the emotions of the subjects. The use of both “Valence-arousal faces” and “5-faces” widgets (further referred as “emospace” and “emoscale”, respectively) was analysed in order to establish whether they are suitable for this purpose.

### 4.1. Co-Validation of Both Widgets

**Figure 6:**
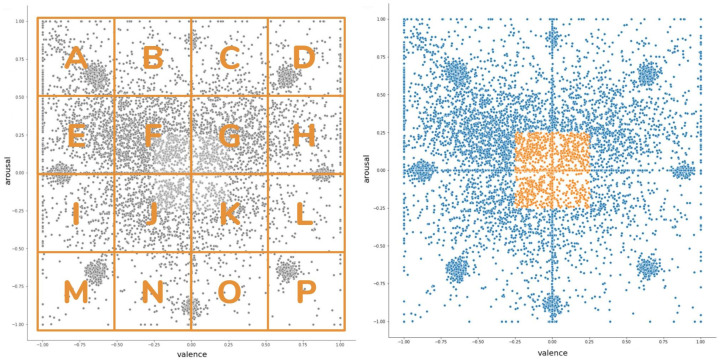
The split of ratings in the emospace widget into clusters (**left**). Cluster _0_ introduced as an intercept for emospace-related analyses (**right**).

Visual inspection of emospace results (see Figure 6) led to the following observations:

- As expected, more extreme emotions appeared less frequently and the ones that were closer to neutrality occurred more often.
- The respondents often chose the coordinates where emoticons are located, which may suggest that the widget was not fully understood by everyone.

In order to compare responses from emospace widget with the rating form emoscale, we arbitrarily divided the two dimensional space into 16 separate clusters (A–P), each covering the one fourth of available arousal and valence vectors (see Figure 6). Next, the intercept of data was stated as an emergent “_0_” cluster (see Figure 6) covering the central area (one-fourth of the available valence and arousal scales).

Based on the trend we observed when comparing both widgets, we assumed that the emoscale levels should be assigned to the emospace clusters, as presented in the “assumed” column in Table 1. We counted the number of assignments of the specific stimuli pairs for each of five levels of emoscale and for each of the emospace clusters (A–P). Subsequently, for each pair, the most frequently assigned level and cluster was selected. The frequency of co-assigning every cluster with every rating revealed the pattern presented in the actual column in Table 1 (for full frequency assignment, see Table A1 in the Appendix A).

### 4.2. Validation of both Widgets Using IADS and IAPS Sets

Two analyses were carried out in order to investigate the reasonableness of widgets responses to the IAPS and IADS stimuli. The first one considered the binary condition (consistent or inconsistent), while the second one considered the specific condition (*p+s+*, p0s0, *p–s–*, *p+s–*, and *p–s+*).

Regarding the binary approach, the following model corresponds to the hypothesis tested:

(2) $$model:widget\;ans_{i}∼B_{0}+B_{1}*binary\;stim\;condition+\left(B_{0}|random\;effects\right)+\epsilon_{i}$$

**Figure 7:**
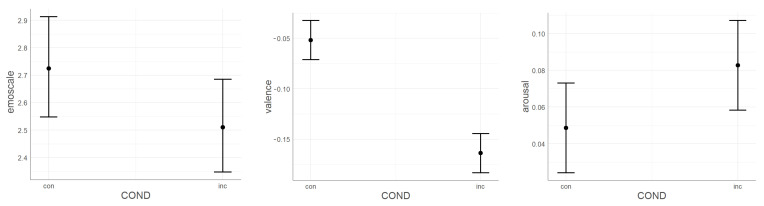
Mixed model of the influence of binary stimuli conditions on: the emoscale widget response (**left**), the emospace valence vector response (**center**), and the emospace arousal vector response (**right**).

**Figure 8:**
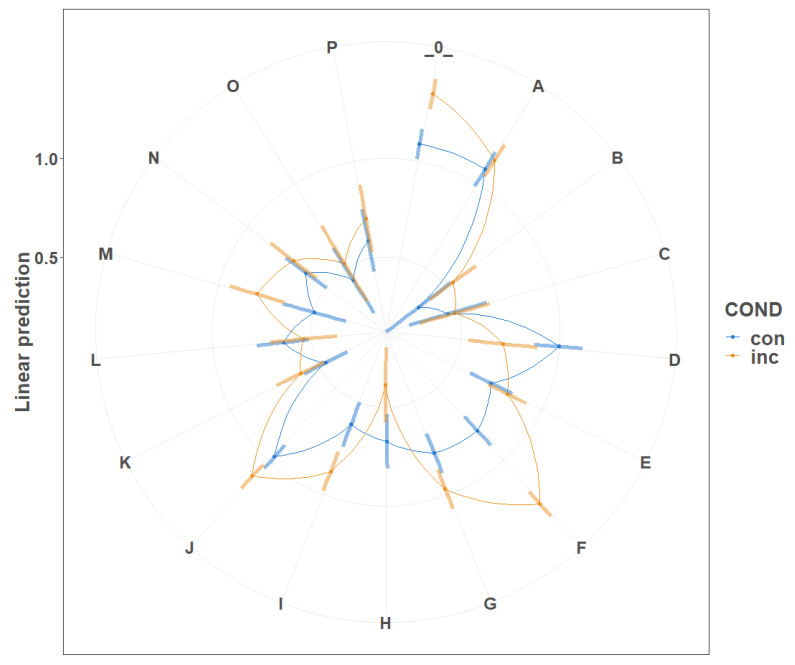
Mixed model of binary stimuli conditions on the emospace widget response.

The emoscale widget response showed a significant effect (Deviance=29,986.588, Figure 7) when compared to the null model (Deviance=30,016.289, Table A2 in the Appendix B). Additionally, in the emospace widget responses, we observed the above-mentioned significant dependence (Deviance=16,756.03, Figure 8) as compared to the null model (Deviance=17,522.63) and the model without interaction (Deviance=16,841.145, Table A3 in the Appendix B).

When considering responses separately on two vectors, valence (Deviance=14,992.09, Figure 7) and arousal (Deviance=11,764.65, Figure 7) both performed better when comparing to the null models (Deviance=15,045.29, Table A4 in the Appendix B and Deviance=11,784.88, Table A5 in the Appendix B, respectively).

In the specific approach, the following model corresponds to the hypothesis tested:

(3) $$model:widget\;answer_{i}∼B_{0}+B_{1}*specific\;stimuli\;condition+\left(B_{0}|random\;effects\right)+\epsilon_{i}$$

**Figure 9:**
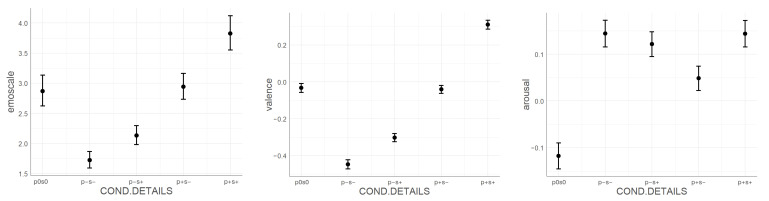
Mixed model of the influence of specific stimuli conditions on: the emoscale widget response (**left**), the emospace valence vector response (**center**), and the emospace arousal vector response (**right**).

**Figure 10:**
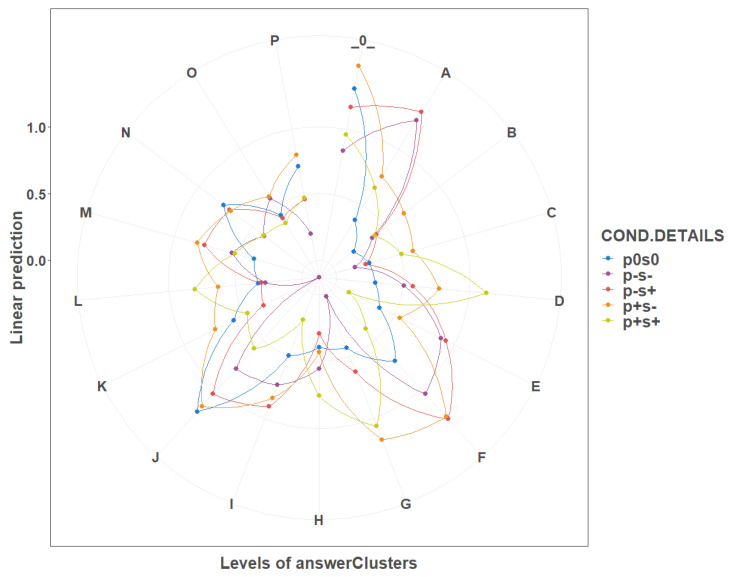
Mixed model of the influence of specific stimuli conditions on the emospace widget response.

Emoscale widget response showed significant effect (Deviance=29,692.40, Figure 9) as compared to the null model (Deviance=30,016.29, Table A6 in the Appendix B). Additionally, in the emospace widget responses, we observed the above mentioned significant dependence and the interaction effect (Deviance=16,318.18, Figure 10), compared to the null model (Deviance=17,522.63) and the model without interaction (Deviance=16,834.28, Table A7 in the Appendix B).

When separately considering responses on two vectors, in both valence and arousal, we observed significant effects. Valence (Deviance=11,609.90, Figure 9) performed better when comparing to the null model (Deviance=15,045.29, Table A8 in the Appendix B). Regarding the arousal model (Deviance=11,150.17, Figure 9), it also performed better when comparing to the null model (Deviance=11,785.316, Table A9 in the Appendix B).

### 4.3. Validation of both Widgets Using Psychophysiological Reactions

In this analysis, we wanted to search for connections between bodily responses and subjective widget answers. The following model corresponds to the hypothesis tested:

(4) $$model:biosignal\;responses_{i}∼B_{0}+B_{1}*widget\;answer+\left(B_{0}|random\;effects\right)+\epsilon_{i}$$

We observed significant effects when only considering the arousal vector of the emospace widget response. Not only the mean RR interval (Deviance=95,777.24), but also GSR response latency (Deviance=94,687.03), are towering over the null counterparts (Deviance=95,781.50, Table A10 and Deviance=94,699.10, Table A11 in the Appendix C, respectively). Both bodily responses increase values with the increasing arousal, based on the emospace widget responses.

### 4.4. Discussion on Widgets Validation

Analyses of the use of both widgets indicate that they are good means of interaction with users. Emospace requires further minor improvements, such as moving the emoticons out of the selection space to remove the bias that is associated with selecting emoticon coordinates (compare Figure 2 and Figure 6).

For binary and specific conditions, data analysis indicates that the consistence of the stimuli pairs on the affective dimension is well recognized. Emospace widget response captures the difference best in the clusters laying on the ascending diagonal of valence-arousal space. Nevertheless, regarding the comparison with psychophysiological reactions, only the arousal dimension in the emospace effectively expresses the bio-markers of emotions.

Because of the completely different nature of both widgets, further analysis will be carried out on emotions that are considered in three different ways:

- emoscale widget responses,
- emospace widget responses (clustered), and
- emospace vector widget responses (arousal and valence vectors, separately).

Interestingly, as it is indicated by the specific approach analysis, the picture of negative valence seems to overpower the positive sound that comes with it (*p–s+* condition), presenting comparable results as the stimuli pair of consistent negative valence (*p–s–* condition). Additionally, the positive valence of the picture cancels out the negative character of sound in pair (*p+s–* condition), which brings the results down to the level of neutral stimuli pair (p0s0 condition). The presented conclusions are particularly interesting from a cognitive perspective. They could potentially be of use in the process of designing affective interfaces which serve to induce the appropriate emotional state or modulate existing stimuli affective scores.

## 5. Personality vs. Simple Audio-Visual Stimuli

IWe first evaluated whether there are relationships between different levels of personality traits and reactions (both widget responses and psychophysiological reactions) to simple affective stimuli in order to verify the main hypothesis of this paper, i.e., whether personality can be used for the emotional adaptation of intelligent systems.

### 5.1. Relation between Widget Responses and Personality Traits

We intended to establish the connections between subjective widget answers and participants’ personality. The following model corresponds to the hypothesis tested:

(5) $$model:widget\;answer_{i}∼B_{0}+B_{1}*personality\;assess.+\left(B_{0}|random\;eff.\right)+\epsilon_{i}$$

The model showed a significant effect of conscientiousness (Deviance=10,721.50) personality axis on emospace widget response in arousal dimension. The proposed fit presents itself as more parsimonious than the one describing the null hypothesis model (Deviance=10,727.36, Table A12 in the Appendix D), with the conscientiousness result decreasing with increasing values of answers on arousal dimension of emospace widget.

**Figure 11:**
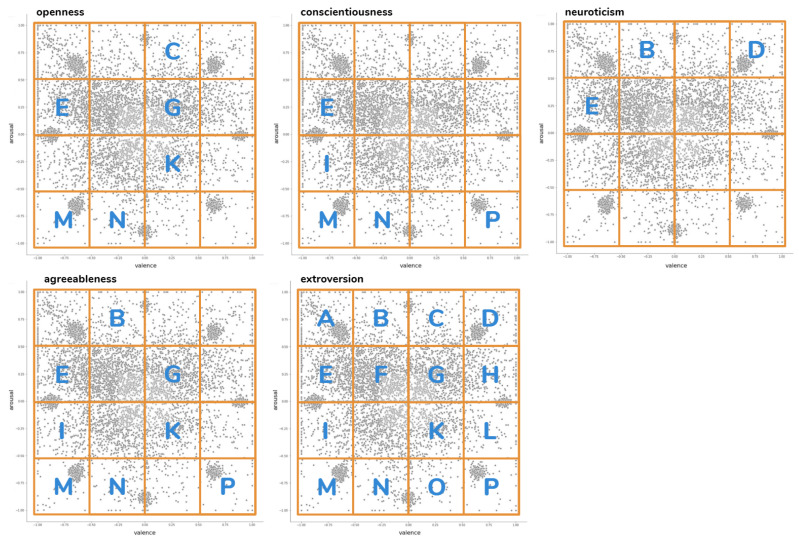
Multinomial logistic regression models concerning the clustered emospace widget responses.

Multinomial logistic regression models concerning the clustered emospace widget responses showed several interesting patterns (see Figure 11):

- For the conscientiousness, the most noticeable differences were spread among the boundary negative arousal (M, N, P) and boundary negative valence (E, I, M) clusters.
- For the agreeableness, the most noticeable differences were spread similarly, among the boundary negative arousal (M, N, P) and negative valence (E, I, M) clusters, with the additional contribution of B, G and K clusters.
- For the extroversion, all but one cluster (J) presented noticeable differences.
- For the neuroticism, the most noticeable differences were spread among the boundary positive arousal (B, D, E).
- For the openness, the pattern of differences was more chaotic and thus left uninterpreted.

All of the multinomial logistic regression models described above are presented in Table A13, Table A14, Table A15, Table A16 and Table A17 in the Appendix D.

### 5.2. Relation between Psychophysiological Reactions and Personality Traits

Analyses were conducted in order to answer the question whether there are relationships between the gathered bio-markers of emotions and the personality. The following model corresponds to the hypothesis tested:

(6) $$model:biosignal\;responses_{i}∼B_{0}+B_{1}*personality\;assess.+\left(B_{0}|random\;eff.\right)+\epsilon_{i}$$

Among a variety of used bio-markers and the five NEO-FFI personality traits, only the heart rate (HR) presented considerable usage in targeting the participant’s personality profile. Openness showed a strong effect on HR measured during the exposure to affective stimuli (Deviance=124,616.58) contrasted with the null version (Deviance=139,729.37, Table A18 in the Appendix E). HR increases together with the tendency for the openness.

### 5.3. Discussion on Personality vs. Simple Audio-Visual Stimuli

In this section, we aimed to establish possible connections between personality traits that were captured using NEO-FFI questionnaire and the responses to simple sound and picture stimuli from IADS and IAPS sets. We were looking for the patterns that could help us to optimize the emotion prediction, while using the biosignals and subjective ratings. Comparisons show that the conscientiousness dimension co-changes well with the subjective arousal rating. The level of conscientiousness decreases with the increasing tendency for the subjective rating of arousal. At the same time, the openness trait co-changes with the heart-rate mean value, as an objective measure of arousal. Openness tendency increases with the increase of heart rate.

The analysis of the response clusters in the emospace widget showed that personality traits present different patterns of response in the valence-arousal space, with the conscientiousness and agreeableness being strongly highlighted in the negative valence and arousal boundaries, and neuroticism evidently contrasted on the positive edge of the arousal dimension.

## 6. Personality vs. Complex Stimuli (Games)

An analysis of personality relationships was undertaken in response to more complex stimuli, which here took the form of a controlled game environment, as significant relationships between personality and responses to simple stimuli were found. In such an environment, the whole context was stored in logs, which were the basis of the described analyses.

### 6.1. Analysis

A series of MANOVAs were conducted with five personality traits (each on one of three levels: high, medium, low) that were gathered by NEO-FFI as independent variables, and with several game-related statistics gathered from game logs as dependent variables.

For Affective SpaceShooter 2 the following values were calculated:

- total number of shots fired (SFN),
- total number of all asteroids destroyed (ADN),
- total number of affective asteroids in positive picture/positive sound condition destroyed to total number of asteroids destroyed ratio (p+s+R),
- total number of affective asteroids in negative picture/negative sound condition destroyed to total number of asteroids destroyed ratio (p−s−R),
- total number of affective asteroids in positive picture/negative sound condition destroyed to total number of asteroids destroyed ratio (p+s−R), and
- total number of affective asteroids in negative picture/positive sound condition destroyed to total number of asteroids destroyed ratio (p−s+R).

Two variables used for Freud me out 2:

- enemies killed to enemies spawned ratio (EKtSR) and
- total number of shots fired (SFN).

There were also other values calculated, e.g., total number of player’s deaths or total number of enemies killed, although we obtained no significant results for them.

The results are presented in Table 2 and Table 3 for Affective SpaceShooter 2 and Freud me out 2, respectively. In order to shorten the tables, only p<0.1 results were reported in them. Significant associations were examined further by non-parametric testing with Tukey’s HSD (see Table A19, Table A20 and Table A21 in the Appendix F).

### 6.2. Discussion on Personality vs. Complex Stimuli (Games)

Several significant associations between game activities and personality traits are a promising result. It indicates that personality can be useful in assessing emotions, not only with simple stimuli, such as movies [33], but also in a more complex experimental environment, like games. The most interesting seems to be the extroversion, generally connected to the increase of activity in game (i.e., number of shots, number of killed enemies). At the same time, it is now clear to us that the better understanding of those relations calls for the better game design—one that would allow us to capture the more nuance effects. In the future work, we are planning to log the game more densely and increase our control over the aims and goals of the player.

## 7. Conclusions

The aim of this paper was to investigate the possibility of using personality assessment as an affective adaptation mechanism in intelligent systems. We were particularly interested in the use of emotion measurement through affordable wearable sensors. We used two affective games of our own as an experimental environment, which allowed for us to present complex stimuli under the complete control of the whole context.

We reported on the results of a large experiment that we conducted to collect the affective data set, called BIRAFFE [42]. Unlike two existing datasets, ASCERTAIN [31] and AMIGOS [32], which also collect affective data along with the personality assessment, BIRAFFE provides details of changes in stimuli over time in a form of game logs, which facilitates the analysis of subjects emotions in reaction to the complex affective stimuli presented. In addition, this study has several more features that distinguish it from existing affective data sets [17,26], such as: a large sample (206 individuals), the inclusion of conflicting stimuli (*p+s–* and *p–s+*), the use of affective games as an experimental environment, and the introduction of new widgets for the self-assessment of emotions by the subjects.

We discovered several significant links between different personality traits, as measured by the NEO-FFI questionnaire, and the characteristics of psychophysiological reactions (ECG, GSR). These are in line with the existing research [33]. Furthermore, we discovered links between personality traits and reactions to complex stimulus environment (games). The above-mentioned results are promising, and they indicate the potential for the adaptation of intelligent systems’ with the use of wearable sensors that we used in our experiments. We believe that the further investigation of those relations will allow for us to introduce personality profiles as an effective tool for the pre-usage adaptation of games and, ultimately, intelligent systems in general.

Determining the type of personality at the beginning of the device usage, e.g., with the use of a personality stories methodology [39], will allow for the prediction of expected user reactions and the proper adjustment of the user interface. This will be made possible by the analysis of game logs, as there are correlations between personality and user behavior. The calibration phase is still required for personalization for a particular person. However, thanks to the pre-coded knowledge regarding different types of personality, it will be shorter and more accurate, as the system will be initially adapted to the specific personality profile. One of the Automatic Personality Recognition methods can also be used if it is feasible to collect relevant data [40]. Consequently, the user will start using the device more quickly. This can also be used in an opposite direction. Users can take a short session with the device, during which psychophysiological signals and usage logs will be collected. Analysis of the data will potentially allow for a rough estimate of personality type without the need for filling out the personality questionnaire. We do not assume that the questionnaire can be replaced by a physiological measurement. However, it is not possible to carry out such a reliable personality measurement when working with a mobile system. Physiological measurement may be a sufficient approximation for such applications.

Moreover, to be able to gain data regarding subjects’ self-assessment more efficiently, we made an attempt to improve the subjective rating tools available, introducing and evaluating our own widgets. The emospace widget turned out to be successive in rating the simple audio-visual stimuli. The personality itself, being our main object of focus, presented promising relations to gathered subjective ratings. As a side outcome, we also presented an analysis that indicates that the image valence dominates over the sound valence. This conclusion can be potentially useful in affective human–computer interfaces design.

This study has potential limitations. The sample was taken from a very narrow demographic range—most of the subjects were students of a technical university. This should not be a big drawback, because physiological reactions and personality are fairly universal. There have also been various technical problems with data recording, so we do not provide complete data for every participant. However, the data set contains a summary of information that is available for each subject [42]. Additionally, our methodology uses games as an example of context-aware intelligent systems. No attempts to use this methodology on real IS, e.g., voice assistants, have been made yet. Finally, the study used the BITalino platform, which can be uncomfortable as a wearable. However, we assume that the rapid development of the technology will lead to superior devices upon which the proposed methodology will then be used.

In the future, we will work on our approach to collect different types of user context, including the emotional one, for system adaptation and personalization. We are working on developing different efficient context providers. As a part of the context analysis, we are also considering the analysis of non-atomic emotional states, i.e., the situation where the person can be in more than one unique emotional state. When considering the games application, we are planning to log the game more densely and increase our control over the aims and goals of a gamer. We will also prepare a catalog of dependencies between the user and the game environment, which can be formalized while using decision rules. The model will then be used to achieve user’s interaction with the system in the “affective” feedback loop.

## Figures and Tables

**Table 1:** A comparison of emoscale ratings and emospace clusters.

Emoscale	Emospace Clusters	
	Assumed	Actual
1	A	A
2	F + J	F + J
3	F + J + G + K	J
4	G + K	G
5	D	D + L

**Table 2:** Selected MANOVA results for variance in Affective SpaceShooter 2 statistics by NEO-FFI results.

	df	SS	MS	F	p
Openness					
p−s+R	2	3.26 × $10^{-4}$	1.63 × $10^{-4}$	3.83	0.03
Conscientiousness					
p+s+R	2	3.50 × $10^{-4}$	1.75 × $10^{-4}$	2.61	0.08
p−s−R	2	2.97 × $10^{-4}$	1.49 × $10^{-4}$	2.57	0.08
Extroversion					
SFN	2	3.98 × $10^{6}$	1.99 × $10^{6}$	5.38	0.006
ADN	2	14,265.20	7132.60	5.09	0.009

Note. Game statistics: *SFN*, *ADN*, p+s+R, p−s+R, p−s−R, are defined in the text.

**Table 3:** Selected MANOVA results for variance in Freud me out 2 statistics by NEO-FFI results.

	df	SS	MS	F	p
Extroversion					
EKtSR	2	0.12	0.06	2.79	0.07
SFN	2	8.41 × $10^{6}$	4.21 × $10^{6}$	6.02	0.004

Note. Game statistics: *EKtSR* and *SFN* are defined in the text.

## Data Availability

The collected dataset is publicly available at Zenodo under CC BY-NC-ND 4.0 license (http://doi.org/10.5281/zenodo.3442143). The scripts used to carry out the presented analyses are available upon request from the corresponding author.

## Abbreviations

The following abbreviations are used in this manuscript:

AfC	Affective Computing
AfCAI	Affective Computing with Context Awareness for Ambient Intelligence
AI	Artificial Intelligence
APR	Automatic Personality Recognition
CAS	Context-Aware Systems
ECG	Electrocardiogram
GSR	Galvanic Skin Response
HR	Heart Rate
IS	Intelligent Systems
NEO-FFI	NEO Five Factor Inventory
SAM	Self-Assessment Mankin

## Appendix A. Co-Validation of Both Widgets

**Table A1:** The frequency of emoscale ratings and emospace clusters co-assignment.

Emoscale Rating	Emospace Cluster	Count
1	A	16
1	E	3
2	A	6
2	E	1
2	F	18
2	J	20
3	F	5
3	G	1
3	J	16
4	D	4
4	F	2
4	G	16
4	H	1
4	J	4
4	K	3
4	L	2
5	D	1
5	L	1

## Appendix B. Validation of Both Widgets Using IADS and IAPS Sets

**Table A2:** Mixed model of the influence of binary stimuli conditions on the emoscale widget response.

	Null Model					Expanded Null Model					Model				
**Predictors**	**Estimate**	***SE***	***CI***	***t***	***p***	**Estimate**	***SE***	***CI***	***t***	***p***	**Estimate**	***SE***	***CI***	***t***	***p***
consistent: _0_	2.71	0.01	[2.66, 2.76]	106.35	<0.001	2.62	0.03	[2.45, 2.80]	28.39	<0.001	2.72	0.03	[2.55, 2.91]	29.32	<0.001
inconsistent											0.92	0.02	[0.89, 0.95]	−5.46	<0.001
*Random Effects*															
$\sigma^{2}$	0.31					0.32					0.32				
$\tau_{00}$	0.01*_ID_*					0.01*_ID_*					0.01*_ID_*				
						0.08*_IAPS_._ID_*					0.08*_IAPS_._ID_*				
*df*	9478					9477					9476				
*N*	158*_ID_*					158*_ID_*					158*_ID_*				
						80*_IAPS_._ID_*					80*_IAPS_._ID_*				
*Deviance*	31,775.62					30,016.29					29,986.59				

**Table A3:** Mixed model of the influence of binary stimuli conditions on the emospace widget response.

	β	*SE*	*z* Value	*p*	*CI*
consistent: _0_	1.086	0.039	27.532	<0.001	[1.009, 1.164]
inconsistent	0.260	0.047	5.549	<0.001	[0.168, 0.353]
B	−0.760	0.108	−7.013	<0.001	[−0.973,−0.548]
C	−0.640	0.107	−5.971	<0.001	[−0.850,−0.430]
E	−0.376	0.066	−5.644	<0.001	[−0.506,−0.245]
F	−0.286	0.057	−5.037	<0.001	[−0.398,−0.175]
G	−0.306	0.060	−5.110	<0.001	[−0.424,−0.189]
H	−0.411	0.075	−5.453	<0.001	[−0.559,−0.263]
I	−0.468	0.067	−6.980	<0.001	[−0.599,−0.336]
J	−0.122	0.048	−2.535	0.011	[−0.216,−0.027]
K	−0.625	0.068	−9.237	<0.001	[−0.757,−0.492]
L	−0.444	0.074	−5.978	<0.001	[−0.589,−0.298]
M	−0.591	0.089	−6.637	<0.001	[−0.766,−0.417]
N	−0.462	0.071	−6.477	<0.001	[−0.602,−0.322]
O	−0.657	0.103	−6.381	<0.001	[−0.859,−0.455]
P	−0.494	0.087	−5.682	<0.001	[−0.665,−0.324]
inconsistent:A	−0.197	0.079	−2.506	0.012	[−0.354,−0.043]
inconsistent:D	−0.544	0.114	−4.768	<0.001	[−0.768,−0.320]
inconsistent:F	0.226	0.075	2.994	0.003	[0.078, 0.373]
inconsistent:H	−0.548	0.125	−4.369	<0.001	[−0.793,−0.302]
inconsistent:L	−0.366	0.115	−3.169	0.001	[−0.593,−0.140]
	σ	SD	Deviance		
ID	0.064	0.253	16,756.03		

**Table A4:** Mixed model of the influence of binary stimuli conditions on the emospace valence vector response.

	Null Model					Model				
**Predictors**	**Estimate**	***SE***	***CI***	***t***	***p***	**Estimate**	***SE***	***CI***	***t***	***p***
consistent: _0_	−0.11	0.01	[−0.13, −0.09]	−12.11	<0.001	−0.05	0.01	[−0.07, −0.03]	−5.18	<0.001
inconsistent						−0.11	0.01	[−0.13, −0.09]	−12.82	<0.001
*Random Effects*										
$\sigma^{2}$	0.22					0.21				
$\tau_{00}$	0.01*_ID_*					0.01*_ID_*				
*df*	11,297					11,296				
*N*	158*_ID_*					158*_ID_*				
*Deviance*	15,045.29					14,882.09				

**Table A5:** Mixed model of the influence of binary stimuli conditions on the emospace arousal vector response.

	Null Model					Model				
**Predictors**	**Estimate**	***SE***	***CI***	***t***	***p***	**Estimate**	***SE***	***CI***	***t***	***p***
consistent: _0_	0.07	0.01	[0.04, 0.09]	5.54	<0.001	0.05	0.01	[0.02, 0.07]	3.90	<0.001
inconsistent						0.03	0.01	[0.02, 0.05]	4.50	<0.001
*Random Effects*										
$\sigma^{2}$	0.16					0.16				
$\tau_{00}$	0.02*_ID_*					0.02*_ID_*				
*df*	11,297					11,296				
*N*	158*_ID_*					158*_ID_*				
*Deviance*	11,784.88					11,764.65				

**Table A6:** Mixed model of the influence of specific stimuli conditions on the emoscale widget response.

	Null Model					Expanded Null Model					Model				
**Predictors**	**Estimate**	***SE***	*CI*	***t***	***p***	**Estimate**	***SE***	***CI***	***t***	***p***	**Estimate**	***SE***	***CI***	***t***	***p***
consistent: _0_	2.71	0.01	[2.66, 2.76]	106.35	<0.001	2.62	0.03	[2.45, 2.80]	28.39	<0.001	2.87	0.05	[2.62, 3.13]	23.33	<0.001
p–s–											0.60	0.06	[0.53, 0.68]	−8.46	<0.001
p–s+											0.74	0.06	[0.66, 0.83]	−5.09	<0.001
p+s–											1.03	0.06	[0.92, 1.15]	0.44	0.662
p+s+											1.33	0.06	[1.19, 1.50]	4.94	<0.001
*Random Effects*															
$\sigma^{2}$	0.31					0.32					0.32				
$\tau_{00}$	0.01*_ID_*					0.01*_ID_*					0.01*_ID_*				
						0.08*_IAPS_._ID_*					0.04*_IAPS_._ID_*				
*df*	9478					9477					9473				
*N*	158*_ID_*					158*_ID_*					158*_ID_*				
						80*_IAPS_._ID_*					80*_IAPS_._ID_*				
*Deviance*	31,775.62					30,016.29					29,692.40				

**Table A7:** Mixed model of the influence of specific stimuli conditions on the emospace widget response.

	β	*SE*	*z* Value	*p*	*CI*
consistent: _0_	1.309	0.052	25.319	<0.001	[1.208, 1.410]
p–s–	−0.472	0.086	−5.484	<0.001	[−0.641,−0.303]
p–s+	−0.139	0.071	−1.959	0.050	[−0.279,0.000]
p+s–	0.172	0.063	2.711	0.007	[0.048, 0.296]
p+s+	−0.350	0.077	−4.571	<0.001	[−0.500,−0.200]
A	−0.931	0.152	−6.140	<0.001	[−1.228,−0.634]
B	−1.116	0.211	−5.298	<0.001	[−1.529,−0.703]
C	−1.046	0.264	−3.962	<0.001	[−1.563,−0.528]
D	−1.018	0.196	−5.196	<0.001	[−1.402,−0.634]
E	−0.935	0.136	−6.869	<0.001	[−1.202,−0.668]
F	−0.594	0.093	−6.391	<0.001	[−0.776,−0.412]
G	−0.872	0.109	−8.002	<0.001	[−1.086,−0.659]
H	−0.916	0.172	−5.331	<0.001	[−1.253,−0.579]
I	−0.811	0.115	−7.063	<0.001	[−1.036,−0.586]
K	−0.723	0.097	−7.434	<0.001	[−0.913,−0.532]
L	−0.979	0.135	−7.235	<0.001	[−1.244,−0.714]
M	−0.930	0.146	−6.357	<0.001	[−1.217,−0.643]
N	−0.541	0.091	−5.965	<0.001	[−0.718,−0.363]
O	−0.891	0.133	−6.718	<0.001	[−1.151,−0.631]
P	−0.592	0.117	−5.069	<0.001	[−0.820,−0.363]
p–s–:A	1.353	0.175	7.732	<0.001	[1.010, 1.696]
p–s+:A	1.089	0.168	6.490	<0.001	[0.760, 1.418]
p+s+:A	0.632	0.262	2.408	0.016	[0.118, 1.146]
p–s–:B	0.647	0.270	2.396	0.017	[0.118, 1.176]
p+s+:B	0.551	0.287	1.919	0.055	[−0.012,1.114]
p+s+:C	0.601	0.293	2.048	0.041	[0.026, 1.176]
p+s+:D	1.186	0.214	5.548	<0.001	[0.767, 1.605]
p–s–:E	0.989	0.167	5.918	<0.001	[0.662, 1.317]
p–s+:E	0.694	0.158	4.411	<0.001	[0.386, 1.004]
p–s–:F	0.808	0.134	6.043	<0.001	[0.546, 1.069]
p–s+:F	0.729	0.119	6.125	<0.001	[0.496, 0.962]
p+s–:F	0.394	0.115	3.433	<0.001	[0.169, 0.619]
p–s+:G	0.332	0.151	2.197	0.028	[0.036, 0.627]
p+s–:G	0.566	0.131	4.315	<0.001	[0.309, 0.823]
p+s+:G	0.979	0.138	7.083	<0.001	[0.708, 1.250]
p+s+:H	0.712	0.197	3.620	<0.001	[0.326, 1.098]
p–s–:I	0.709	0.153	4.640	<0.001	[0.409, 1.008]
p–s+:I	0.547	0.142	3.853	<0.001	[0.269, 0.825]
p+s–:J	−0.227	0.093	−2.444	0.014	[−0.409,−0.045]
p+s+:J	−0.286	0.123	−2.326	0.020	[−0.528,−0.045]
p+s+:L	0.823	0.167	4.939	<0.001	[0.497, 1.150]
p–s–:M	0.643	0.195	3.304	<0.001	[0.262, 1.025]
p–s+:M	0.524	0.181	2.894	0.004	[0.169, 0.880]
p–s–:O	0.623	0.275	2.264	0.024	[0.084, 1.163]
	σ	SD	Deviance		
ID	0.066	0.256	16,318.18		

**Table A8:** Mixed model of the influence of specific stimuli conditions on the emospace valence vector response.

	Null Model					Model				
**Predictors**	**Estimate**	***SE***	***CI***	***t***	***p***	**Estimate**	***SE***	***CI***	***t***	***p***
consistent: _0_	−0.11	0.01	[−0.13, −0.09]	−12.11	<0.001	−0.03	0.01	[−0.06, −0.01]	−2.73	0.006
p–s–						−0.41	0.01	[−0.44, −0.39]	−31.79	<0.001
p–s+						−0.27	0.01	[−0.29, −0.25]	−22.91	<0.001
p+s–						−0.01	0.01	[−0.03, −0.01]	−0.66	0.509
p+s+						0.34	0.01	[−0.32, −0.37]	26.60	<0.001
*Random Effects*										
$\sigma^{2}$	0.22					0.16				
$\tau_{00}$	0.01_*ID*_					0.01_*ID*_				
*df*	11,297					11,293				
*N*	158_*ID*_					158_*ID*_				
*Deviance*	15,045.29					11,609.90				

**Table A9:** Mixed model of the influence of specific stimuli conditions on the emospace arousal vector response.

	Null Model					Model				
**Predictors**	**Estimate**	***SE***	***CI***	***t***	***p***	**Estimate**	***SE***	***CI***	***t***	***p***
consistent: _0_	0.06	0.02	[0.02, 0.10]	2.86	0.004	−0.12	0.01	[−0.15, −0.09]	−8.29	<0.001
p–s–						0.26	0.01	[0.24, 0.29]	20.56	<0.001
p–s+						0.24	0.01	[0.22, 0.26]	20.79	<0.001
p+s–						0.17	0.01	[0.14, 0.19]	14.81	<0.001
p+s+						0.26	0.01	[0.24, 0.29]	20.74	<0.001
*Random Effects*										
$\sigma^{2}$	0.16					0.15				
$\tau_{00}$	0.02_*ID*_					0.02_*ID*_				
*df*	11,297					11,293				
*N*	158_*ID*_					158_*ID*_				
*Deviance*	11,785.32					11,150.17				

## Appendix C. Validation of Both Widgets Using Psychophysiological Reactions

**Table A10:** Mixed model of the influence of emospace arousal widget response vector results on the mean RR interval.

	Null Model					Model				
**Predictors**	**Estimate**	***SE***	***CI***	***t***	***p***	**Estimate**	***SE***	***CI***	***t***	***p***
(Intercept)	754.87	9.81	[735.65, 774.09]	76.96	<0.001	754.61	9.81	[735.38, 773.84]	76.92	<0.001
arousal						3.61	1.75	[0.18, 7.03]	2.07	0.039
*Random Effects*										
$\sigma^{2}$	4765.72					4763.80				
$\tau_{00}$	15,097.26_*ID*_					15,101.41_*ID*_				
*df*	8397					8396				
*N*	158_*ID*_					158_*ID*_				
*Deviance*	95,781.50					95,777.24				

**Table A11:** Mixed model of the influence of emospace arousal widget response vector results on the GSR response latency.

	Null Model					Model				
**Predictors**	**Estimate**	***SE***	***CI***	***t***	***p***	**Estimate**	***SE***	***CI***	***t***	***p***
(Intercept)	758.23	12.77	[733.20, 783.27]	59.36	<0.001	754.64	12.72	[729.72, 779.56]	59.35	<0.001
arousal						48.56	13.98	[21.17, 75.95]	3.47	0.001
*Random Effects*										
$\sigma^{2}$	231,809.10					231,479.01				
$\tau_{00}$	19,195.20_*ID*_					18,811.63_*ID*_				
*df*	6216					6215				
*N*	155_*ID*_					155_*ID*_				
*Deviance*	94,699.10					94,687.03				

## Appendix D. Relation between Widget Responses and Personality Traits

**Table A12:** Mixed model of the influence of conscientiousness on the emospace widget response in the arousal dimension.

	Null Model					Model				
**Predictors**	**Estimate**	***SE***	***CI***	***t***	***p***	**Estimate**	***SE***	***CI***	***t***	***p***
(Intercept)	0.06	0.01	[0.04, 0.09]	4.93	<0.001	0.13	0.03	[0.07, 0.19]	4.21	<0.001
conscientiousness						−0.01	0.01	[−0.02, −0.00]	−2.43	0.01
*Random Effects*										
$\sigma^{2}$	0.16					0.16				
$\tau_{00}$	0.02_*ID*_					0.02_*ID*_				
*df*	10158					10,157				
*N*	142_*ID*_					142_*ID*_				
*Deviance*	10,727.36					10,721.50				

**Table A13:** Emospace widget response multinomial logistic regression model for agreeableness concerning the space of a widget as clusters.

Odds Ratio	*SE*	*CI*	*t*	*p*	Response
0.99	0.02	[0.96, 1.03]	−0.51	0.609	A
0.92	0.03	[0.87, 0.97]	−3.00	0.003	B
0.97	0.03	[0.91, 1.04]	−0.85	0.395	C
1.02	0.02	[0.98, 1.07]	1.04	0.297	D
1.05	0.02	[1.01, 1.09]	2.51	0.012	E
1.01	0.02	[0.98, 1.05]	0.76	0.446	F
1.06	0.02	[1.02, 1.10]	2.89	0.004	G
1.05	0.03	[1.00, 1.11]	1.90	0.058	H
0.94	0.02	[0.91, 0.98]	−3.10	0.002	I
1.01	0.02	[0.98, 1.04]	0.86	0.389	J
1.07	0.02	[1.02, 1.12]	2.99	0.003	K
0.99	0.02	[0.95, 1.04]	−0.30	0.763	L
0.92	0.03	[0.87, 0.96]	−3.40	0.001	M
0.89	0.02	[0.85, 0.93]	−5.31	<0.001	N
1.01	0.03	[0.94, 1.08]	0.25	0.805	O
0.92	0.03	[0.87, 0.97]	−2.94	0.003	P
*Deviance*	53,096.36				

**Table A14:** Emospace widget response multinomial logistic regression model for extroversion concerning the space of a widget as clusters.

Odds Ratio	*SE*	*CI*	*t*	*p*	Response
1.13	0.02	[1.09, 1.17]	6.64	<0.001	A
1.09	0.03	[1.03, 1.15]	2.90	0.004	B
1.13	0.03	[1.06, 1.20]	3.56	<0.001	C
1.14	0.02	[1.09, 1.20]	5.52	<0.001	D
1.08	0.02	[1.03, 1.12]	3.58	<0.001	E
1.05	0.02	[1.02, 1.09]	2.85	0.004	F
1.09	0.02	[1.04, 1.13]	4.17	<0.001	G
1.15	0.03	[1.09, 1.21]	5.21	<0.001	H
1.12	0.02	[1.08, 1.17]	5.71	<0.001	I
1.03	0.02	[0.99, 1.06]	1.56	0.120	J
1.06	0.02	[1.01, 1.10]	2.37	0.018	K
1.14	0.03	[1.08, 1.19]	4.97	<0.001	L
1.10	0.03	[1.05, 1.16]	3.76	<0.001	M
1.17	0.02	[1.11, 1.22]	6.53	<0.001	N
1.25	0.03	[1.17, 1.34]	6.54	<0.001	O
1.13	0.03	[1.06, 1.19]	4.15	<0.001	P
*Deviance*	53,074.55				

**Table A15:** Emospace widget response multinomial logistic regression model for neuroticism concerning the space of a widget as clusters.

Odds Ratio	*SE*	*CI*	*t*	*p*	Response
1.00	0.02	[0.98, 1.03]	6.64	0.762	A
1.08	0.02	[1.03, 1.13]	2.90	0.002	B
1.04	0.03	[0.98, 1.10]	3.56	0.169	C
1.05	0.02	[1.01, 1.09]	5.52	0.019	D
1.05	0.02	[1.01, 1.09]	3.58	0.005	E
1.00	0.01	[0.97, 1.03]	2.85	0.912	F
0.98	0.02	[0.95, 1.01]	4.17	0.237	G
1.02	0.02	[0.98, 1.07]	5.21	0.396	H
1.01	0.02	[0.98, 1.05]	5.71	0.490	I
0.99	0.01	[0.97, 1.02]	1.56	0.567	J
1.00	0.02	[0.96, 1.04]	2.37	0.968	K
1.00	0.02	[0.96, 1.05]	4.97	0.907	L
1.00	0.02	[0.96, 1.04]	3.76	0.969	M
0.98	0.02	[0.94, 1.02]	6.53	0.260	N
1.02	0.03	[0.96, 1.08]	6.54	0.565	O
0.98	0.02	[0.94, 1.03]	4.15	0.498	P
*Deviance*	53,185.50				

**Table A16:** Emospace widget response multinomial logistic regression model for openness concerning the space of a widget as clusters.

Odds Ratio	*SE*	*CI*	*t*	*p*	Response
1.01	0.02	[0.97, 1.05]	0.05	0.618	A
1.03	0.03	[0.97, 1.10]	0.95	0.343	B
1.08	0.04	[1.00, 1.17]	2.06	0.040	C
0.97	0.03	[0.92, 1.03]	−0.95	0.343	D
1.08	0.02	[1.03, 1.13]	3.19	0.001	E
1.04	0.02	[1.00, 1.08]	1.82	0.069	F
1.08	0.02	[1.04, 1.13]	3.61	<0.001	G
1.03	0.03	[0.97, 1.09]	0.87	0.383	H
1.01	0.02	[0.97, 1.06]	0.62	0.537	I
1.00	0.02	[0.96, 1.03]	−0.12	0.905	J
1.06	0.03	[1.01, 1.12]	2.45	0.014	K
0.98	0.03	[0.93, 1.04]	−0.53	0.599	L
1.06	0.03	[1.00, 1.13]	2.08	0.037	M
1.08	0.03	[1.03, 1.14]	2.95	0.003	N
1.07	0.04	[0.99, 1.15]	1.69	0.091	O
1.05	0.03	[0.98, 1.11]	1.39	0.166	P
*Deviance*	53,175.81				

**Table A17:** Emospace widget response multinomial logistic regression model for conscientiousness concerning the space of a widget as clusters.

Odds Ratio	*SE*	*CI*	*t*	*p*	Response
0.98	0.02	[0.95, 1.02]	−1.09	0.275	A
0.92	0.03	[0.86, 0.97]	−2.95	0.003	B
0.98	0.03	[0.91, 1.04]	−0.70	0.484	C
0.96	0.02	[0.91, 1.01]	−1.70	0.090	D
0.93	0.02	[0.89, 0.97]	−3.64	<0.001	E
1.01	0.02	[0.97, 1.04]	0.34	0.735	F
1.02	0.02	[0.98, 1.06]	0.92	0.356	G
0.96	0.02	[0.91, 1.01]	−1.61	0.108	H
0.96	0.03	[0.92, 1.00]	−2.06	0.039	I
1.00	0.03	[0.97, 1.03]	−0.14	0.887	J
1.01	0.02	[0.96, 1.05]	0.30	0.766	K
0.97	0.02	[0.92, 1.02]	−1.36	0.173	L
1.09	0.03	[1.04, 1.15]	3.35	0.001	M
1.08	0.02	[1.03, 1.13]	3.13	0.002	N
1.97	0.04	[0.90, 1.03]	−0.98	0.326	O
1.14	0.03	[1.08, 1.21]	4.51	<0.001	P
*Deviance*	53,124.38				

## Appendix E. Relation between Psychophysiological Reactions and Personality Traits

**Table A18:** Mixed model of the influence of openness on the HR.

	Null Model					Model				
**Predictors**	**Estimate**	***SE***	***CI***	***t***	***p***	**Estimate**	***SE***	***CI***	***t***	***p***
(Intercept)	−0.28	1.21	[−2.65, 2.09]	−0.23	0.818	−7.57	3.45	[−14.32, −0.81]	−2.20	0.028
openness						1.32	0.61	[0.11, 2.52]	2.14	0.032
*Random Effects*										
$\sigma^{2}$	114.77					111.51				
$\tau_{00}$	222.48_*ID*_					210.77_*ID*_				
*df*	18,319					16,399				
*N*	153_*ID*_					137_*ID*_				
*Deviance*	139,729.37					124,616.58				

## Appendix F. Personality vs. Complex Stimuli (Games)

**Table A19:** Tukey Post Hoc Results of SFN and ADN statistic (SpaceShooter 2) by NEO-FFI Extroversion trait (see Table 2 for MANOVA results).

Extroversion Level	*SD*	*M*	1.	2.
SFN				
1. High	559.09	940.48		
2. Medium	616.96	949.50	174.82 [−268.79, 618.44]	
3. Low	606.24	1394.94	582.30 [65.73, 1098.87]	−407.48 [−811.76, −3.20]
ADN				
1. High	44.50	317.22		
2. Medium	38.30	327.39	9.83 [−17.69, 37.35]	
3. Low	17.18	353.17	36.13 [4.08, 68.17]	−26.30 [−51.38, −1.22]

Note. *M* indicates mean. *SD* indicates standard deviation. Values in square brackets indicate the 95% confidence intervals.

**Table A20:** Tukey Post Hoc Results of p−s+R statistic (SpaceShooter 2) by NEO-FFI Openness trait (see Table 2 for MANOVA results).

Openness Level	*SD*	*M*	1.	2.
1. High	0.007	0.03		
2. Medium	0.007	0.03	−0.0005 [−0.006, 0.005]	
3. Low	0.006	0.03	0.004 [−0.002, 0.01]	−0.005 [−0.009, 0.000]

Note. *M* indicates mean. *SD* indicates standard deviation. Values in square brackets indicate the 95% confidence intervals.

**Table A21:** Tukey Post Hoc Results of SFN statistic (Freud me out 2) by NEO-FFI Extroversion trait (see Table 3 for MANOVA results).

Extroversion Level	SD	*M*	1.	2.
1. High	60.65	149.6		
2. Medium	59.6	141.2	−60.13 [−649.93, 529.68]	
3. Low	39.93	185.53	718.92 [25.21, 1412.63]	−779.04 [−1327.96, −23014]

Note. Values in square brackets indicate the 95% confidence intervals.
